# Relationships between longitudinal neutrophil to lymphocyte ratios, body weight changes, and overall survival in patients with non-small cell lung cancer

**DOI:** 10.1186/s12885-017-3122-y

**Published:** 2017-02-16

**Authors:** B. A. Derman, J. N. Macklis, M. S. Azeem, S. Sayidine, S. Basu, M. Batus, F. Esmail, J. A. Borgia, P. Bonomi, M. J. Fidler

**Affiliations:** 1 Rush University Medical Center, Chicago, IL USA; 2 University of Texas Southwestern Medical Center, Dallas, TX USA; 3 Department of Internal Medicine, 1717 W Congress Parkway, 1025 Kellogg, Chicago, IL 606012 USA; 4 Division of Hematology/Oncology, 1725 W. Harrison St., Suite 809, Chicago, IL 60612 USA; 5 Division of Hematology/Oncology, 5323 Harry Hines Boulevard, Dallas, TX 75390 USA; 6 Department of Pathology, 1750 W. Harrison St., Suite 1415, Chicago, IL 60612 USA; 7 Department of Internal Medicine, 1717 W. Congress Parkway, 10 Kellogg, Chicago, IL 60612 USA

**Keywords:** NSCLC, Weight gain, Neutrophil to lymphocyte ratio, Cancer cachexia

## Abstract

**Background:**

There is emerging evidence showing a significant relationship between overall survival (OS) in non-small cell lung cancer NSCLC patients and weight change during chemotherapy or chemoradiation. A high neutrophil/lymphocyte ratio (NLR) at baseline and at follow-up is associated with shorter survival in cancer patients and may be a surrogate for ongoing inflammation, implicated in cancer cachexia and tumor progression. The objective of this study is to explore potential relationships between OS, serial weights, and serial NLRs in advanced NSCLC patients receiving chemotherapy.

**Methods:**

One hundred thirty-nine patients with chemotherapy-naïve NSCLC, predominantly with stage III/IV disease, were treated with first-line platinum doublets from June, 2011 to August, 2012. NLR, tumor response, and body weight were recorded at baseline, 6, and 12 weeks from initiation of therapy and correlated with OS. The association between NLR and OS was assessed using Cox PH (proportional hazards) analysis, the association between NLR and weight change was assessed using a simple regression analysis, and the association between NLR and tumor response was assessed using the Fisher’s exact test.

**Results:**

One hundred thirty-nine patients with median age 68, PS 0-1/2 = 83/17%, male/female = 58%/42%. Median NLR at baseline was 3.6 (range 0.1898 to 30.910), at 6 weeks 3.11 (range 0.2703 to 42.11), and at 12 weeks 3.52 (range 0.2147 to 42.93). A Higher NLR at baseline, 6, and 12 weeks was associated with decreased OS (baseline: HR 1.06, *p* < 0.001; 6 weeks: HR 1.07, *p* = 0.001; 12 weeks: HR 1.05, *p* < 0.001), and longitudinal NLR, as a time-dependent covariate, was also associated with decreased OS (HR = 1.06, *p* < 0.001). Baseline weight and NLR were inversely related (cor = −0.267, *p* = 0.001), and weight change and NLR were inversely related at 12 weeks (cor = −0.371, *p* < 0.001). Longitudinal measurements of weight and NLR were also negatively associated (slope = −0.06, *p* < 0.001). Using a cutoff of NLR > 5, there was a significant association between progressive disease and NLR > 5 at 6 weeks (*p* = 0.02) and 12 weeks (*p* = 0.03).

**Conclusions:**

High baseline and progressive increases in NLRs are associated with progressive disease, inferior OS and weight loss in NSCLC patients. In addition to having prognostic significance, these observations suggest that studying molecular mediators of cachexia/inflammation and their relationships to tumor progression may identify new therapeutic targets in the large subset of NSCLC patients who have cancer cachexia.

## Background

Cancer cachexia represents a multifactorial spectrum of disease that has been defined by international consensus as “an ongoing loss of skeletal muscle mass (with or without loss of fat mass) that cannot be fully reversed by conventional nutritional support and leads to progressive functional impairment” [1]. Prevalence rates of cachexia differ by malignancy type, with approximately 60% of NSCLC patients experiencing it [2]. Cancer cachexia may be a result of both reduced nutritional intake and increased resting energy expenditure [3]. More recent evidence suggests that there are several other factors that contribute to cancer cachexia, which includes increased insulin resistance, hypogonadism, adrenergic activation, and activation of proinflammatory responses [4]. Identifying and combating this phenomenon has taken on great importance in light of studies showing that weight change in advanced NSCLC patients receiving concurrent chemoradiation or chemotherapy alone is inversely associated with overall survival (OS) [5–7]. Similarly, loss of muscle mass (regardless of BMI) is also associated with worse functional status and OS [8].

The NLR is the ratio between the neutrophil and lymphocyte counts; what constitutes an elevated value varies between >3.5 and >5. Several studies have already shown that an increased baseline NLR is associated with poor clinical outcomes for several types of cancers, including NSCLC [9–13]. There is relatively little information regarding longitudinal NLRs, which some have posited may be even more predictive of individual patient outcomes [14] and may be a dynamic indicator of tumor status, cachexia, and ongoing inflammation. Our objective was to investigate potential relationships between longitudinal NLRs, body weights, and overall survival.

## Methods

One hundred thirty-nine patients with NSCLC who were treated with first-line platinum doublets from June, 2011 to August, 2012 were reviewed for this study; none of the patients received prior therapy with an EGFR tyrosine kinase inhibitor. 127 patients had stage III or stage IV NSCLC, and 12 patients had either stage I or stage II disease and received adjuvant chemotherapy following surgical resection. This study was approved by the institutional review board, and compliant with the Helsinki declaration. NLR and body weight were recorded at baseline, 6, and 12 weeks from initiation of therapy and correlated with OS. All patients had blood drawn in outpatient clinic on the day of evaluation and treatment for advanced NSCLC. None had active infections at the time of these visits. Neutrophil and lymphocyte counts were measured on the Sysmex XN-9000 Hematology Analyzer, Sysmex Corporation, Kobe, Japan. Albumin was measured on the Architect Clinical Chemistry Analyzer C16000, Abbott Diagnostics, Santa Clara, California. The association of OS at NLR at baseline, 6 weeks, and 12 weeks was assessed using the Kaplan-Meier method, the log-rank test, and the Cox proportional hazards (PH) analysis. The effect of serial NLR measured longitudinally on OS was assessed using a time-dependent covariate Cox PH analysis. The longitudinal association between weight and NLR was measured serially at baseline, 6 and 12 weeks was assessed using a mixed effects longitudinal analysis. The association between NLR and tumor response was assessed using a Fisher’s exact test. Tumor response rates were calculated for the patients with measurable disease according to the RECIST (Response Evaluation Criteria in Solid Tumors) 1.1 guidelines. The ALI index was defined as the BMI*Albumin/NLR, and ALI was calculated at baseline, 6 and 12 weeks from initiation of therapy and correlated with OS; 96, 93, and 84 patients had ALI scores available at baseline, 6 and 12 weeks respectively.

## Results

Of 139 patients evaluated, the median age was 68 years, and 58% were male. 83% had a performance score of 0–1 and 17% had a performance score of 2 (Table 1).

**Table 1:** Demographics

Age at Diagnosis (years)		
	Median	68
	Mean	65
Gender (n, %)		
	Female	58 (42%)
	Male	81 (58%)
Ethnicity (n, %)		
	Caucasian	94 (67.6%)
	African American	35 (25.2%)
	Hispanic	6 (4.3%)
	Asian	3 (2.2%)
	Other	1 (0.7%)
Smoking History At Diagnosis (n, %)		
	Current Smoker	56 (40.3%)
	Former Smoker	72 (51.8%)
	Never Smoker	11 (7.91%)
Stage at Diagnosis (n, %)		
	Stage I^a^	2 (1.4%)
	Stage II^a^	10 (7.2%)
	Stage III	54 (38.9%)
	Stage IV	72 (51.8%)
	Other	1 (0.7%)
Histopathology (n, %)		
	Adenocarcinoma	92 (66.2%)
	Squamous Cell	42 (30.2%)
	Large Cell	2 (1.4%)
	Other	3 (2.2%)
Performance Status (n, %)		
	0	44 (31.6%)
	1	71 (51.1%)
	2	21 (15.1%)
	3	3 (2.2%)
	4	0 (0%)

^a^Received adjuvant chemotherapy following surgical resection

The median NLR at baseline (Table 2) was 3.6 (range 0.1898 to 30.910), at 6 weeks 3.11 (range 0.2703 to 42.11), and at 12 weeks 3.52 (range 0.2147 to 42.93). Higher NLR at baseline, 6 and 12 weeks were associated with decreased OS (baseline: HR 1.06, *p* < 0.001; 6 weeks: HR 1.07, *p* = 0.001; 12 weeks: HR 1.05, *p* < 0.001). When the serial measurements of NLR measured longitudinally at baseline, 6 and 12 weeks are considered as a time-dependent covariate, a Cox PH analysis continued to support its strongly significant association with decreased OS (HR = 1.06, *p* < 0.001).

**Table 2:** Neutrophil-to-Lymphocyte Ratios at Baseline, 6 Weeks & 12 Weeks

Interval	Change in NLR (n, %)	Median NLR	Range
Baseline	–	3.6	0.1898 – 30.910
6 Weeks	Increase (63, 45.6%)	3.11	0.2703 – 42.11
	Decrease (75, 54.4%)		
	N/A (1)		
12 Weeks	Increase (67, 51.9%)	3.52	0.2147 – 42.93
	Decrease (62, 48.1%)		
	N/A (10)		

**Fig. 1:**
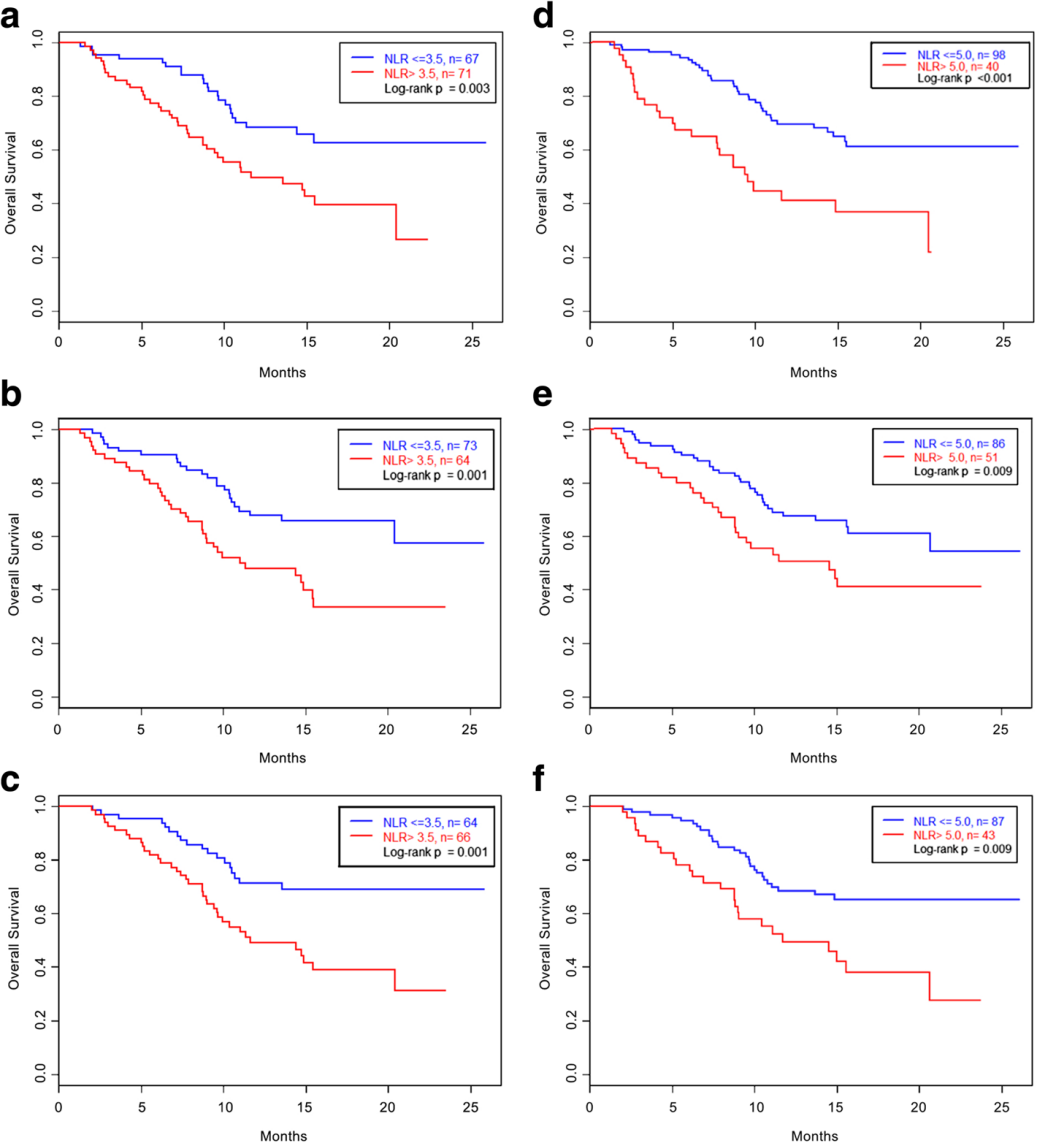
Kaplan Meier Survival Plots by NLR. Kaplan Meier survival plots were generated using an NLR cutoff of 3.5 at baseline (**a**), at 6 weeks (**b**), and at 12 weeks (**c**) and an NLR cutoff of 5 at baseline (**d**), at 6 weeks (**e**), and at 12 weeks (**f**). A significant difference in overall survival was seen in all three categories, illustrating that an NLR > 3.5 and an NLR > 5 serve as poor prognostic factors associated with worse overall survival at diagnosis and during chemotherapy

NLR > 3.5 and NLR > 5 have both been used to define the elevated NLR group [15]. As shown in Fig. 1, there were strongly significant differences in OS between the elevated (>3.5) and non-elevated (≤3.5) NLR groups at baseline (NLR ≤ 3.5 group median OS not reached vs. NLR >3.5 group median OS 11.6 months, *p* = 0.003), at 6 weeks (median OS not reached vs 11.4 mos, *p* = 0.001) and at 12 weeks (median OS not reached vs 11.6 mos, *p* = 0.001). The same held true when using an NLR cutoff of 5. The median OS in the non-elevated (≤5) NLR group at baseline, 6 weeks, and 12 weeks were not reached; the median OS in the elevated (>5) NLR group at baseline was 9.08 months, at 6 weeks was 11 months, and at 12 weeks was 11 months (*p* ≤ 0.009).

Baseline weight and baseline NLR were negatively correlated (cor = −0.267, *p* = 0.001), and the change in weight from baseline to 12 weeks and 12 week-NLR were also inversely related (cor = −0.371, *p* < 0.001). The serial measurements of weight and NLR were found to be significantly associated in a longitudinal mixed effects regression analysis (slope = −0.06, *p* < 0.001).

The range of weight change was −13.17 kg to +16.61 kg (median −0.5 kg, mean −0.89 kg) over 12 weeks from initiation of chemotherapy. 55 patients experienced weight gain ≥ +0.1 kg at 12 weeks; of that group, 41 patients exhibited weight gain at 6 weeks. Of the 55 patients who gained weight at 12 weeks, the median weight gain was +2.41 kg. 73 patients lost weight at 12 weeks, and the median weight loss was −3.08 kg (Table 3). 37 patients gained albumin at 12 weeks; of that group, 25 patients had gained albumin at 6 weeks. The median albumin gain was +0.4 g/dL. 40 patients had a decrease in albumin at 12 weeks, with a median loss of −0.2 g/dL. 20 patients had no change in their albumin (Table 3). Although there is variability amongst individual patients, there is a longitudinal inverse relationship between NLR and weight change (cor −0.06224, *p* < 0.001). There was no observed association between weight change and albumin change at 6 or 12 weeks (*p* = 0.265).

**Table 3:** Changes in Weight and Albumin at 6 & 12 Weeks

Weight Change at 6 Weeks (n)	Weight Change at 12 Weeks (n)	Median Weight Change at 12 Weeks
Increase (55)	Increase (55)	+2.41 kg
Decrease (80)	Decrease (73)	−3.08 kg
No change (4)	No change (1)	0 kg
	N/A (10)	
Albumin Change at 6 Weeks (n)	Albumin Change at 12 Weeks (n)	Median Albumin Change at 12 Weeks
Increase (36)	Increase (37)	+0.4 g/dl
Decrease (59)	Decrease (40)	−0.2 g/dl
No Change	No Change (20)	0 g/dl
N/A (44)	N/A (42)	
No. Maintained Weight Gain from Weeks 6 to 12		41/55 (74.5%)
No. Maintained Albumin Gain from Weeks 6 to 12		25/37 (67.6%)

In addition, of the 96 patients who had a baseline ALI score available (Table 4), 38 (39.5%) patients had a baseline ALI < 18 with median OS of 9.6 months compared to the 58 patients with ALI ≥ 18 with median OS not reached (*p* = 0.001). Of 93 patients who had a 6 week ALI score available, 41 (44%) had an ALI < 18 with median OS 11.4 months compared to the 52 patients with ALI >18 with median OS not reached (*p* = 0.03). Of the 84 patients who had a 12 week ALI score available, 30 (35.7%) had an ALI < 18 with median OS of 9 months compared to the 54 patients with ALI ≥ 18 with median OS not reached (*p* < 0.001).

**Table 4:** Advanced Lung Cancer Inflammation Index

	Baseline (*n* = 97)	6 Weeks (*n* = 94)	12 Weeks (*n* = 84)
ALI Range	1.73 - 497.36	0 - 337.16	1.15 - 429.79
ALI Median	21.61	25.02	25.71
ALI <18 Median OS	9.6 Months	11.4 Months	9 Months
ALI ≥18 Median OS	Not Reached	Not Reached	Not Reached
*p*-value	*p* = 0.001	*p* = 0.03	*p* < 0.001

Tumor response was calculated at both 6 and 12-week intervals. In total, there were 85 patients for whom a 6-week response was measurable and 95 patients for whom a 12-week response was measurable. Response data were not available for 54 patients at the 6-week time point and for 44 patients at the 12-week time point because they had evaluable disease only based on RECIST 1.1 response criteria or a CT scan was not performed at that time point. Using a cutoff of NLR > 5, there was a significant association between progressive disease and NLR > 5 at 6 weeks (*p* = 0.02) and at 12 weeks (*p* = 0.03). With a cutoff of NLR > 3.5, there was a significant association between progressive disease and NLR >3.5 at 6 weeks (*p* = 0.02), with a trend toward significance at 12 weeks (*p* = 0.06).

## Discussion

Our understanding of the relationship between cancer cachexia, inflammation, and survival in NSCLC continues to evolve. Pretreatment ALI (BMI*Albumin/NLR) has been one attempt to bridge these three concepts, and was shown to be an independent marker of poor outcome in patients with advanced NSCLC when the ALI < 18 compared to ALI ≥ 18 [16]. Those patients had more sites of metastatic disease, and poorer PFS (2.4 vs. 5.1 months, *p* < 0.001) and OS (3.4 vs. 8.3 months, *p* < 0.001)*.* Our findings here confirm that a pretreatment ALI < 18 is associated with poorer OS in advanced stage NSCLC.

Tumor markers such as carcinoembryonic antigen (CEA), cancer antigen 125 (CA-125), and squamous cell carcinoma (SCC) antigen have been used to assist in diagnosis and prognosis in lung cancer but are not definitive [17, 18]. The advantage of using the NLR alone as a longitudinal prognostic marker is in its ease of use. The ability to use a single calculation to estimate survival, derived only from a complete blood count with differential, is attractive. A recent meta-analysis demonstrated that an elevated NLR is associated with shorter progression free and overall survival in both NSCLC and SCLC, though the authors note the study was limited by bias in publication, selection, and heterogeneity [19]. Another meta-analysis found that the NLR had consistent prognostic value for overall survival in NSCLC with a cutoff value of 5 [20]. In patients receiving first-line epidermal growth factor receptor tyrosine kinase inhibitors (EGFR TKIs), the data are heterogeneous. While one study showed that an elevated NLR ≥ 3.5 was associated with poor outcomes in EGFR-mutated advanced NSCLC [15], another has shown that NLR did not affect survival in EGFR-mutated advanced NSCLC [21].

The data presented here not only shows that higher baseline NLRs are associated with inferior OS, but that progressive increases in NLR during treatment also portend a worse prognosis. Importantly, longitudinal NLRs are inversely related to both overall survival and serial body weights. Thus, the NLR has the potential for prognostication throughout a patient’s treatment course. This is confirmed by the evidence that shows a significant association between progressive disease and NLR > 5 at both 6 and 12 weeks following therapy initiation. These findings suggest that patients with higher systemic inflammation at diagnosis may have more aggressive disease and should be treated promptly and potently, while an increasing NLR during treatment may be a harbinger of disease progression and treatment failure.

Our findings are in line with evidence suggesting the role of neutrophils in modulating the cancer milieu. Neutrophils have the ability to suppress antitumor immunity, promote tumor cell proliferation, and enhance tumor cell survival, all which serve to promote tumor growth. Aging neutrophils are phagocytosed by macrophages, which have the ability to increase vascular permeability that enables tumor cell intravasation and metastasis [22]. Supporting the potential specific association between NLR and survival in NSCLC are studies in animal models with a *STK11/LKB1*-inactivating mutation. Inactivation of this tumor suppressor gene led to significant increases in tumor-promoting cytokines and neutrophils with T-cell suppressive effects. Interestingly, when an IL-6 antibody was introduced to the murine model, the mice experienced both a significant decrease in tumor-associated neutrophils and a significant improvement in survival compared to the control mice [23].

## Conclusions

It is likely that NLR is a surrogate for ongoing inflammation, and that inflammation may be a linchpin that links tumor progression with cachexia and overall survival. While previous studies have shown the prognostic value of baseline NLR, it has been suggested that longitudinal NLRs may be more informative for individual patients [14]. The negative correlation between longitudinal NLRs, overall survival, and body weights observed in our patients suggests that simply measuring longitudinal NLRs may provide important prognostic information and may serve as a real-time indicator of disease progression and the degree of inflammation occurring in individual NSCLC patients. If this observation is confirmed in larger studies, it could have important implications for developing treatments for cancer cachexia and for designing combinations of immune modulators.

## Abbreviations

ALI: Advanced lung cancer inflammation index
BMI: Body mass index
CXI: Cachexia index
EGFR: Epidermal growth factor receptor
NLR: Neutrophil-to-lymphocyte ratio
NSCLC: Non-small cell lung cancer
OS: Overall survival
PH: Proportional hazards
RECIST: Response evaluation criteria in solid tumors
TKI: Tyrosine kinase inhibitor
